# P-1388. How Does Tuberculosis Present in the 21st Century? A Retrospective Cohort Study of Tuberculosis Cases in a Large Academic Health System 2013-2024

**DOI:** 10.1093/ofid/ofaf695.1575

**Published:** 2026-01-11

**Authors:** Isaac H Y Chan, Scott C Roberts, Patrick G T Cudahy

**Affiliations:** Yale School of Medicine, New Haven, CT; Yale School of Medicine, New Haven, CT; Yale School of Medicine, New Haven, CT

## Abstract

**Background:**

Tuberculosis (TB) incidence in the United States (US) has been increasing since 2020. Despite this, understanding of the clinical presentation of TB is largely based on historical case series from the previous century, or from endemic areas. We sought to characterize the initial presentation and clinical characteristics of TB in the 21^st^ century.

**Figure:**
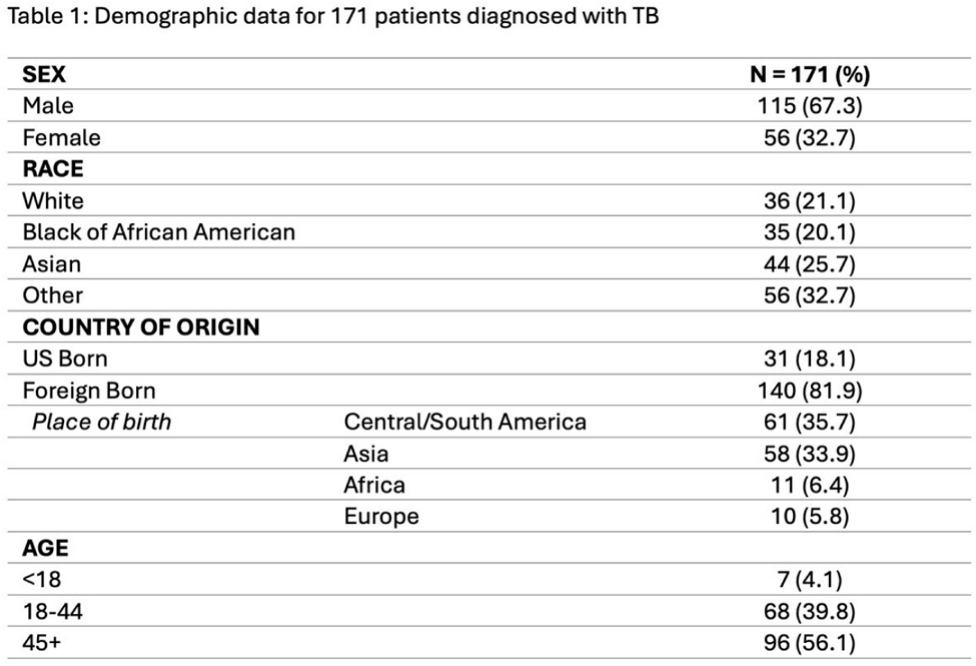

**Figure:**
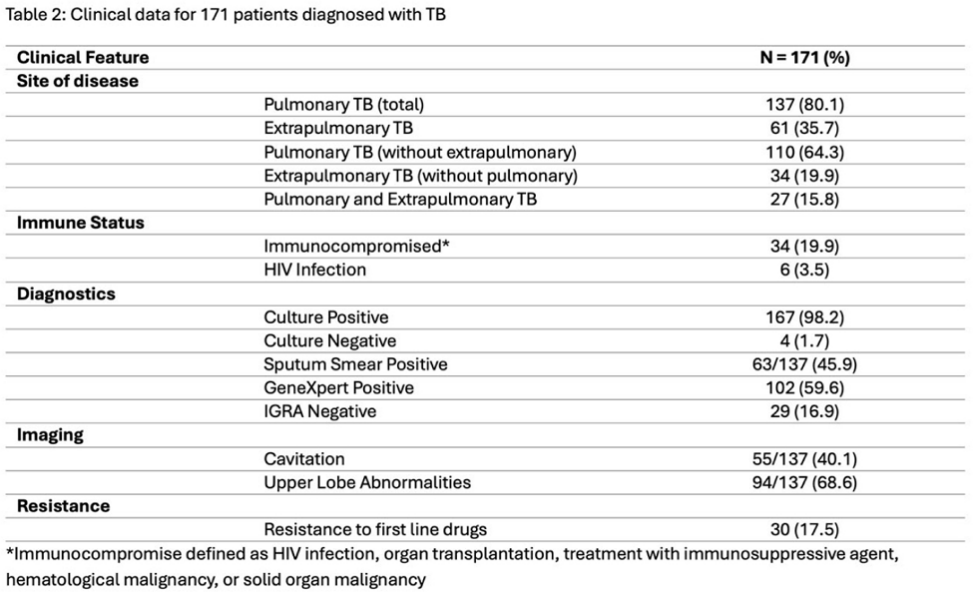

**Methods:**

We reviewed all active TB cases diagnosed at Yale-New Haven Health System labs (inpatient and outpatient) from December 2013 to September 2024. Data was extracted from medical records by the authors.

**Figure:**
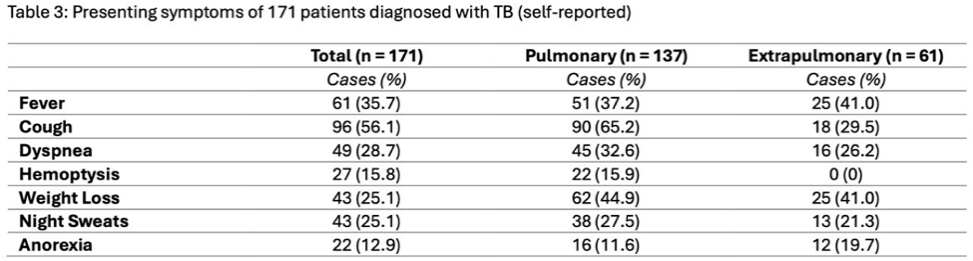

**Figure:**
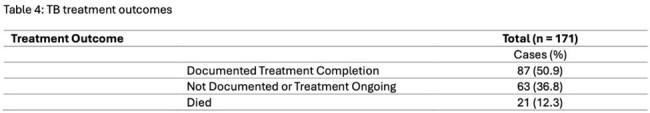

**Results:**

171 active TB cases were identified. 167 cases were culture positive, while 4 cases were culture negative with positive PCR. 115 (67%) were male. 7 cases (4%) were in pediatric patients. 140 (82%) patients were foreign born. There were 137 cases (80%) of pulmonary TB, 61 cases (36%) of extrapulmonary TB, and 27 (16%) cases with both pulmonary and extrapulmonary TB. The most common chief complaint was cough (n = 96, 56%), but no cardinal TB symptom was consistently associated with TB disease. Of the pulmonary TB cases, 55 (40.1%) had radiological evidence of cavitating lesions and 94 (68.6%) had upper lobe abnormalities. 29 (17%) patients had negative or indeterminate interferon-gamma release assay (IGRA). Drug resistance to first-line medications was detected in 30 (17%) cases, 90% of whom were born overseas. 21 (12.3%) patients died prior to completing treatment.

**Conclusion:**

The initial presentation of TB infection in a contemporary US context appears to differ from that in the published literature. Cardinal symptoms of TB are less frequently reported in our patient population compared to historical studies, which may delay TB diagnosis. Despite less symptomatic disease, we noted a higher rate of cavitating disease than previously described, potentially due to increased rates of CT imaging, or a higher proportion of immunocompromised patients. This could also contribute to our observation of a higher rate of concomitant pulmonary and extrapulmonary TB disease than classically understood. IGRA testing continues to be frequently ordered despite its low utility in diagnosing active TB infection. We noted a high TB case fatality rate, which is likely due to host factors in our multimorbid population.

**Disclosures:**

Scott C. Roberts, MD, Pfizer: Advisor/Consultant|Prime Therapeutics: Honoraria|Pri-Med: Honoraria

